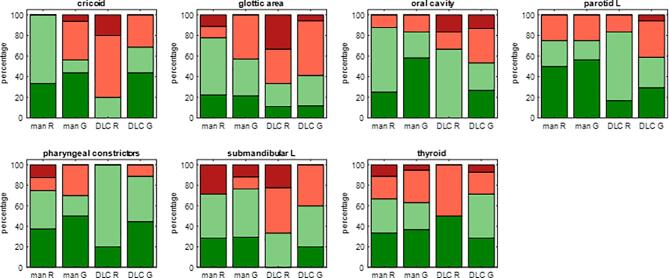# External validation of deep learning-based contouring of head and neck organs at risk

**DOI:** 10.1016/j.phro.2020.06.006

**Published:** 2020-07-10

**Authors:** Ellen J.L. Brunenberg, Isabell K. Steinseifer, Sven van den Bosch, Johannes H.A.M. Kaanders, Charlotte L. Brouwer, Mark J. Gooding, Wouter van Elmpt, René Monshouwer

**Affiliations:** a Department of Radiation Oncology, Radboud University Medical Center, Nijmegen, The Netherlands; b Department of Radiation Oncology, University of Groningen, University Medical Center Groningen, Groningen, The Netherlands; c Mirada Medical Ltd, Oxford, United Kingdom; d Department of Radiation Oncology (MAASTRO), GROW School for Oncology and Developmental Biology, Maastricht University Medical Centre+, Maastricht, The Netherlands

**Keywords:** Deep learning, Auto-contouring, Contour comparison, Head & neck cancer

## Abstract

**Background and purpose:**

Head and neck (HN) radiotherapy can benefit from automatic delineation of tumor and surrounding organs because of the complex anatomy and the regular need for adaptation. The aim of this study was to assess the performance of a commercially available deep learning contouring (DLC) model on an external validation set.

**Materials and methods:**

The CT-based DLC model, trained at the University Medical Center Groningen (UMCG), was applied to an independent set of 58 patients from the Radboud University Medical Center (RUMC). DLC results were compared to the RUMC manual reference using the Dice similarity coefficient (DSC) and 95th percentile of Hausdorff distance (HD95). Craniocaudal spatial information was added by calculating binned measures. In addition, a qualitative evaluation compared the acceptance of manual and DLC contours in both groups of observers.

**Results:**

Good correspondence was shown for the mandible (DSC 0.90; HD95 3.6 mm). Performance was reasonable for the glandular OARs, brainstem and oral cavity (DSC 0.78–0.85, HD95 3.7–7.3 mm). The other aerodigestive tract OARs showed only moderate agreement (DSC 0.53–0.65, HD95 around 9 mm). The binned measures displayed the largest deviations caudally and/or cranially.

**Conclusions:**

This study demonstrates that the DLC model can provide a reasonable starting point for delineation when applied to an independent patient cohort. The qualitative evaluation did not reveal large differences in the interpretation of contouring guidelines between RUMC and UMCG observers.

## 1 Introduction

In image-guided radiotherapy, the amount of data to be segmented is continuously expanding. This is due to multi-modality imaging, adaptive radiotherapy, an increasing number of structures correlated with radiation-induced toxicity, and modern treatment modalities that improve organs-at-risk (OARs) sparing. Automatic OAR delineation can be useful to reduce delineation time and mitigate inter-observer variability [1], [2]. Especially in head and neck (HN) cancer, the potential benefit is substantial. HN delineation is time-consuming because of the complex anatomy with an increasing number of structures added to the delineation, e.g. salivary glands [3], swallowing muscles [4], and carotid arteries [5]. In addition, regular adaptation is necessary as a result of large anatomical variations (i.e. weight loss and tumor shrinkage).

Atlas-based auto-segmentation (ABAS) is routinely used clinically. Although ABAS reduces workload and inter-observer variability, it has its shortcomings. The most important is that only limited anatomical variation can be included (typically 10 to 30 patients), because more would compromise atlas performance in terms of speed [6]. In addition, atlas selection can be an issue [7], even when using a large database [8]. To improve these shortcomings, automatic delineation using deep learning contouring (DLC) is a promising method. Typically, a convolutional neural network (CNN) is used to derive a model from a (large) set of training data. Because of increased computing power, DLC can now be implemented in radiotherapy clinical practice [9].

The added value of DLC has already been shown for different sites, including thorax [10], rectum [11], and liver SBRT [12]. For contouring of HN OARs, van Dijk et al. showed that DLC outperforms ABAS and is almost within the level of inter-observer variability [13]. The authors trained their model on a cohort of 549 HN cancer patients and validated it on an independent cohort of 104 patients. According to the recommendations of Valentini et al. [2] they did not only use geometric and dosimetric measures, but also contouring time, inter-observer variation, and qualitative evaluation.

However, it is not practical, nor desirable for each center to train their own model, and generic solutions for automatic delineation according to agreed international guidelines would be preferable. In addition to saving resources, these would facilitate consistent comparison of radiation-induced side effects between centers. The OARs in Van Dijk et al. [13] were delineated according to international consensus guidelines [14], so the DLC model is potentially widely applicable. Nevertheless, variation in auto-contouring acceptance exists between institutions [15], and the performance of this model on external cases has not been tested yet.

In this study, an independent external validation of the model used by van Dijk et al. [13] was performed on a set of 58 HN cancer patients. DLC contours were evaluated by global and local geometric measures. In addition, we performed a qualitative evaluation, to check for bias in the interpretation of delineation guidelines.

## 2 Materials and methods

**Supplementary figure 1:**
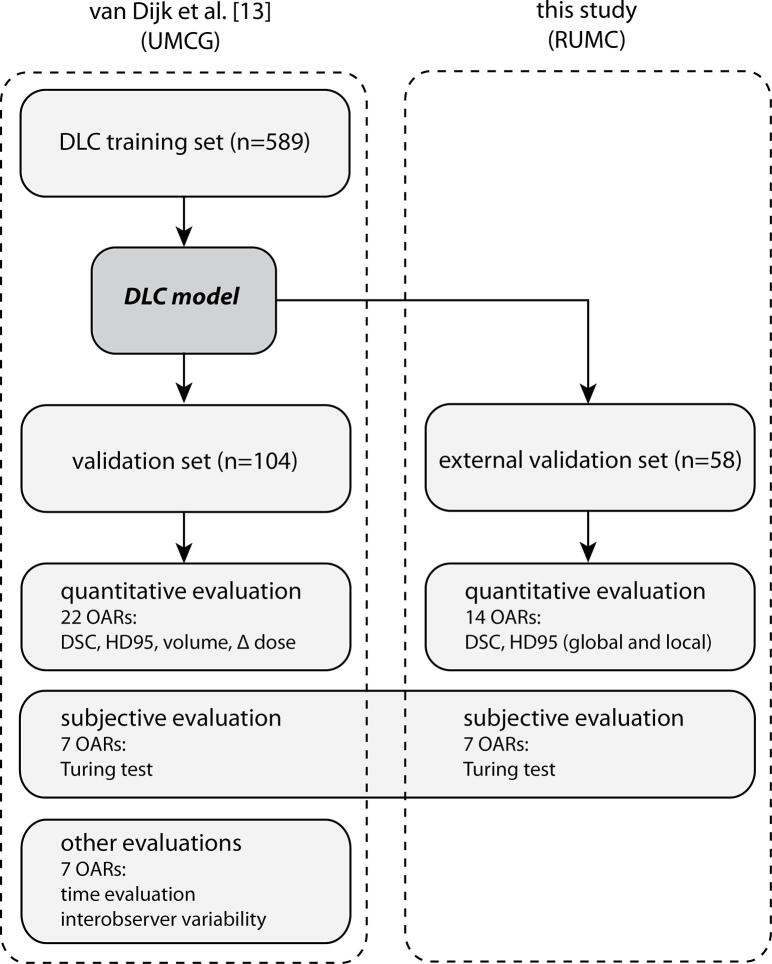


Supplementary Fig. S1 shows an overview of the independent external validation, in relation to Van Dijk et al. [13].

### 2.1 DLC model development

Van Dijk et al. [13] trained their DLC model on data (planning CT and OAR contours) from 589 HN cancer patients treated at the University Medical Center Groningen (UMCG), of which 549 were used for training, while the other 40 were used for cross-validation. Patient characteristics can be found in Table 1. The CT data (average voxel size 0.98 × 0.98 × 2 mm, mostly contrast-enhanced) were acquired on different scanners (Somatom Sensation Open, Somatom Definition AS, or Biograph 64, Siemens, Forchheim, Germany). Manual delineation of the OARs was performed by UMCG expert observers, according to international consensus guidelines [14].

**Table 1:** Overview of patient characteristics, comparing the external validation cohort to the patient population used by Van Dijk et al. (cv = cross validation) [13].

characteristics	validation set		train set [13]		cv set [13]		test set [13]	
	n = 58	%	n = 549	%	n = 40	%	n = 104	%
**sex**								
female	14	24	139	25	13	33	21	20
male	44	76	410	75	27	68	83	80

**age**								
18–65 years	35	60	368	67	20	50	64	62
>65 years	23	40	181	33	20	50	40	38

**tumor site**								
oropharynx	20	34	194	35	15	38	45	43
nasopharynx		0	24	4	3	8	2	2
hypopharynx	7	12	53	10	2	5	10	10
larynx	31	53	255	46	18	45	38	37
oral cavity		0	23	4	2	5	9	9
other		0		0		0		0

Van Dijk et al. [13] considered 22 OARs, divided in 3 sub-groups:

1) glandular: parotid and submandibular glands (left and right), thyroid gland;
2) aerodigestive tract: arytenoids and buccal mucosa (left and right), extended oral cavity, pharynx constrictor muscle, cricopharyngeal inlet (cricoid), supraglottic area, glottic area, cervical esophagus;
3) other: central nervous system, vessels, bone: brainstem, cerebellum, cerebrum, spinal cord, mandible, carotid arteries (left and right).

The DLC implementation was performed with a commercial software package (DLCExpert™, Mirada Medical Ltd., UK). Convolutional neural networks were used to predict labels for input data. The first step consists of a general 2D multiclass network with 14 layers, resulting in a coarse OAR prediction. This prediction, together with the original data, is fed to an OAR-specific 10-layer network that results in a full resolution binary classification [13], [16].

### 2.2 Patients for external validation

For the external validation, a cohort of 58 HN cancer patients was selected from a previous study. This cohort consisted of 44 males and 14 females, divided over three tumor sites: oropharynx (n = 20), hypopharynx (7), and larynx (31). As can be seen in Table 1, the validation cohort was comparable to the training and test sets used by Van Dijk et al. [13]. Patients were treated with primary accelerated radiotherapy according to the UPGRADE-RT protocol [17], delivering 68 Gy in 34 fractions, using a 2-arc VMAT technique. All patients enrolled up to August 2018 at the Radboud University Medical Center (RUMC, Nijmegen, The Netherlands) were selected. Informed consent was acquired, covering this retrospective analysis according to internal review board policy.

All patients underwent planning PET-CT (Biograph 64, Siemens, Forchheim, Germany) with a median resolution of 0.98 × 0.98 × 3 mm (range 0.78 to 1.52 mm in-plane), a peak kilovoltage of 120 kV, and filter kernel I40s\3 for the CT images. Clinical OAR contours, manually delineated on the CT data by RUMC expert radiation oncologists according to the same international consensus guidelines [14], were used as a reference. This reference comprised a subset of fourteen OARs that were delineated consistently in the validation cohort, divided in three groups analogous to [13]:

1) glandular: parotid and submandibular glands (left and right), thyroid gland;
2) aerodigestive tract: buccal mucosa (left and right), extended oral cavity, pharynx constrictor muscle, cricopharyngeal inlet (cricoid), supraglottic area, glottic area;
3) other: brainstem, mandible.

For the external validation, the CT data were processed by the same commercial software package and the model developed by Van Dijk et al. [13]. Comparison between manual delineation and DLC was feasible for 53 patients of the validation set. The other 5 patients were excluded because of incomplete CT data and/or manual reference contours.

### 2.3 Quantitative evaluation

The performance of the DLC in relation to manual contours was evaluated using the Dice similarity coefficient (DSC) and Hausdorff distance (HD). The DSC is a voxel-wise measure of the spatial overlap between two contoured areas *A* and *B* [18], [19]:

$$\mathrm{DSC}\left(A,B\right)=\frac{2\left(A\cap B\right)}{A+B}$$

The bidirectional HD assesses pairwise distances between two contours. For all points *a* on the surface *S_A_* of *A*, the minimum distance *d(a,b)* to points *b* on the surface *S_B_* of *B* are calculated, and vice versa. The HD is the maximum of all minimum distances *d* [18]:

$$HD\left(A,B\right)=\max\left\{\max_{a\in S_{A}}\min_{b\in S_{B}}d\left(a,b\right),\max_{b\in S_{B}}\min_{a\in S_{A}}d\left(b,a\right),\right\}$$

As recommended by Menze et al. [18], in this study the more robust 95th percentile (HD95) was used.

In addition to the global calculation of these two measures, a binned analysis of DSC and HD95 was performed. Contours were divided in four equally spaced bins in the craniocaudal direction, with bin 1 the most caudal and bin 4 the most cranial. In this way, spatial information on the performance of DLC could be obtained.

### 2.4 Qualitative evaluation

To assess the potential influence of different groups of observers, a qualitative evaluation was carried out using a so-called “Turing test”, during which the observer has to determine whether he is interacting with a human or a machine. As explained by Gooding et al. [20], DLC results are considered to be clinically usable if they are difficult to distinguish from manually delineated contours.

In this study, the qualitative evaluation described by Van Dijk et al. [13] was extended with the answers of 4 RUMC observers (2 physicians and 2 technicians involved in OAR contouring for HN cancer patients). The full test comprising 100 questions was completed by 3 of the 4 observers, and in total, 352 observations were generated. More details on this evaluation can be found in the Supplementary Material.

## 3 Results

### 3.1 Quantitative evaluation

**Fig. 1:**
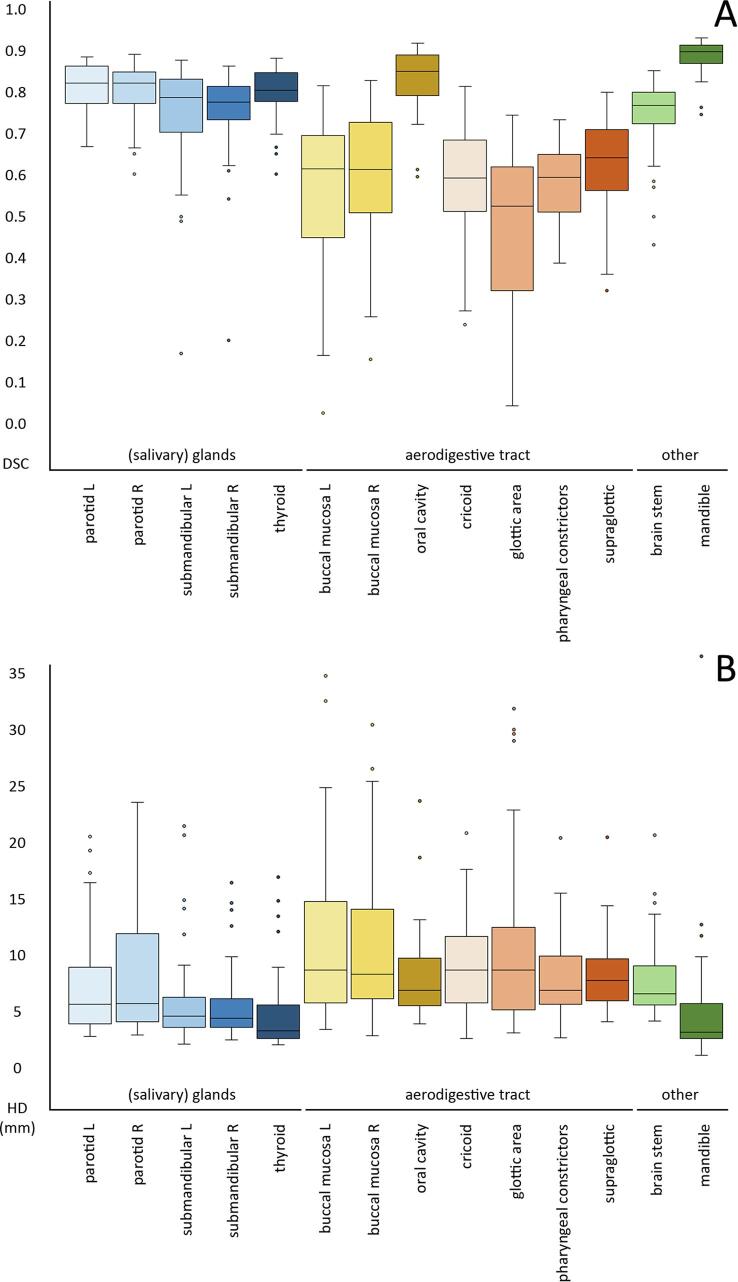
Boxplots of DSC (A) and HD95 (B) for the comparison between DLC and manual delineation for 14 HN OARs in three groups. Glandular OARs: blue; aerodigestive tract: yellow and orange; brainstem and mandible: green. (For interpretation of the references to colour in this figure legend, the reader is referred to the web version of this article.)

**Fig. 2:**
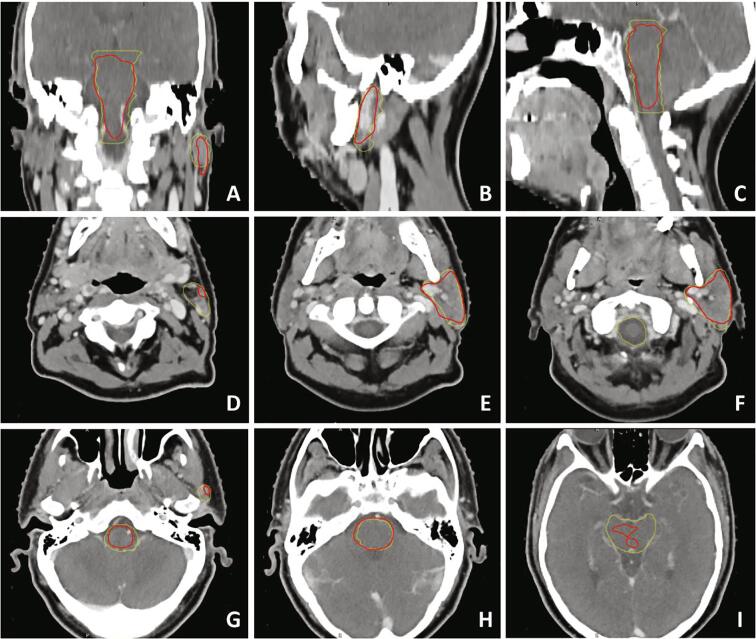
CT slices of an example case, showing differences in performance dependent on craniocaudal location. Contours are displayed for brainstem and left parotid gland, with manual RUMC reference contours given in yellow, and DLC results in red. A: coronal image; B: sagittal image showing left parotid gland contours; C: sagittal image displaying brainstem; D-I: transversal images, with D the most caudal and I the most cranial image. (For interpretation of the references to colour in this figure legend, the reader is referred to the web version of this article.)

*For all glandular OARs*, the DLC model showed borderline good DSC (0.78–0.83) on the RUMC set (cf. Table 2, Fig. 1A). The HD95 was reasonable for submandibular and thyroid glands (3.7–5.0 mm) and slightly larger for the parotid glands (6.0–6.1 mm) (Table 3, Fig. 1B). According to the binned results, the DLC performs well in the middle, but worse on caudal and/or cranial boundaries (see for example the left parotid gland in Fig. 2).

**Table 2:** Median DSC values for the different evaluations of fourteen contoured OAR.

	benchmark from [13]	global DSC	bin 1 (caudal)	bin 2	bin 3	bin 4 (cranial)
**(salivary) glands**						
parotid L	0.85	0.83	0.73	0.87	0.88	0.73
parotid R	0.83	0.83	0.73	0.85	0.86	0.72
submandibular L	0.80	0.79	0.81	0.83	0.79	0.62
submandibular R	0.80	0.78	0.81	0.86	0.79	0.59
thyroid	0.85	0.81	0.80	0.85	0.83	0.66

**aerodigestive tract**						
buccal mucosa L	0.77	0.61	0.52	0.63	0.64	0.57
buccal mucosa R	0.77	0.62	0.49	0.61	0.65	0.60
oral cavity	0.91	0.86	0.78	0.89	0.89	0.84
cricoid	0.72	0.60	0.00	0.76	0.77	0.43
glottic area	0.73	0.54	0.33	0.65	0.60	0.00
pharyngeal constrictors	0.70	0.60	0.60	0.71	0.62	0.54
supraglottic	0.80	0.65	0.51	0.79	0.74	0.24

**other**						
brainstem	0.87	0.78	0.61	0.81	0.87	0.72
mandible	0.95	0.90	0.95	0.91	0.92	0.72

**Table 3:** Median HD95 values (in mm) for the different evaluations of fourteen contoured OARs.

	benchmark from [13]	global HD95	bin 1 (caudal)	bin 2	bin 3	bin 4 (cranial)
**(salivary) glands**						
parotid L	4.2	6.0	6.0	5.0	4.5	6.7
parotid R	4.4	6.1	6.0	5.2	5.3	6.1
submandibular L	3.9	5.0	3.8	4.0	4.6	6.1
submandibular R	4.0	4.8	3.7	3.4	4.3	5.9
thyroid	2.9	3.7	3.9	3.3	3.0	5.8

**aerodigestive tract**						
buccal mucosa L	4.0	9.1	9.4	9.0	7.3	7.8
buccal mucosa R	4.0	8.7	8.4	9.2	7.2	9.0
oral cavity	4.2	7.3	7.1	6.4	6.7	7.7
cricoid	5.0	9.1	9.0	3.9	3.3	6.6
glottic area	3.1	9.1	6.1	5.1	5.8	9.4
pharyngeal constrictors	3.8	7.3	9.1	3.6	5.7	8.3
supraglottic	3.4	8.1	7.1	5.1	4.7	7.4

**other**						
brainstem	3.7	6.9	7.7	4.2	4.2	8.1
mandible	1.2	3.6	2.0	3.3	1.5	8.5

*For the aerodigestive tract OARs*, DSC for the RUMC set was quite low (0.53–0.65), except for the oral cavity (0.85). For the HD95, all OARs in this group resulted in moderately large values (7.3–9.1 mm). The binned DSC and HD95 again showed deviations at caudal and cranial boundaries, especially for the cricoid, glottic area, and supraglottic area. It is worth noting that bin 2 showed the largest deviations for the buccal mucosa ROIs.

*For the brainstem*, DSC for the RUMC set was intermediate to good (0.78), while HD95 was borderline poor (6.9 mm). The binned measures showed the largest differences for the caudal (DSC) and cranial (HD95) bin (see Fig. 2). *For the mandible,* high global correspondence between manual and DLC contours was found (DSC of 0.90, HD95 of 3.6 mm). For both measures, the cranial bin showed the largest local deviations.

### 3.2 Qualitative evaluation

**Fig. 3:**
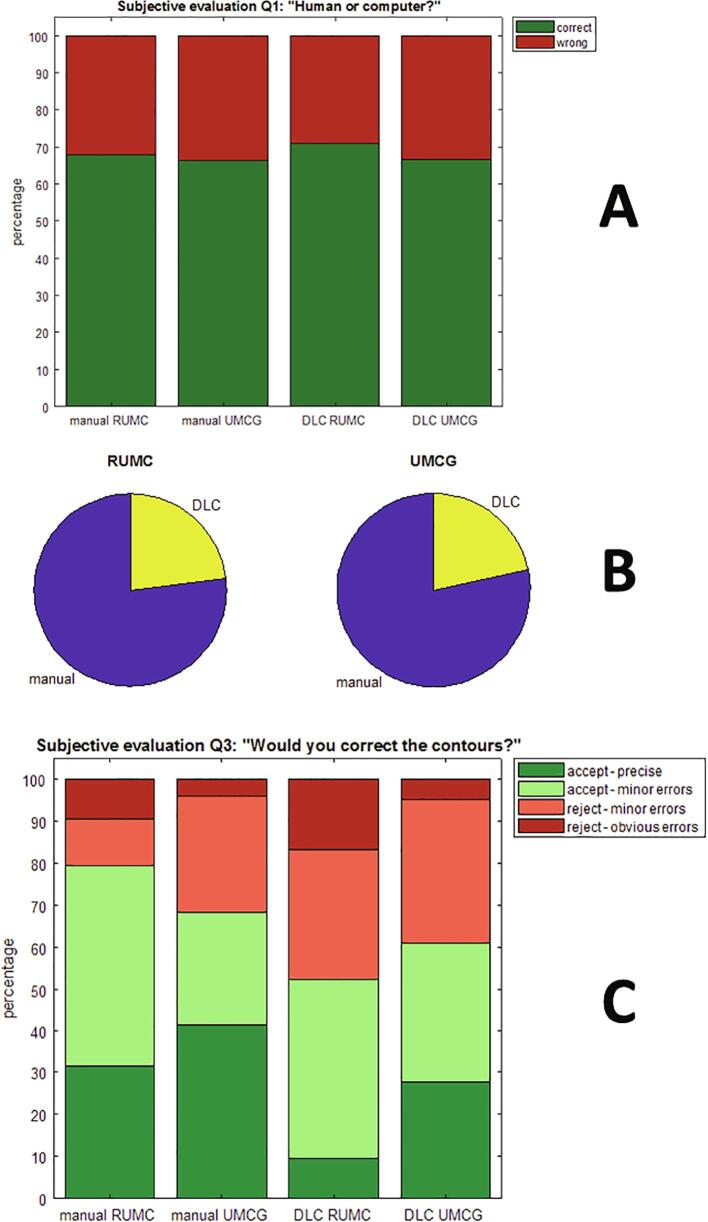
Results of qualitative evaluation. A: Answer to first question of qualitative evaluation on contour source (human or computer). B: Answer to second question: “Which contour do you prefer?”. C: Answer to third question: “Would you correct the contours?” For the bar charts hold that the first two bars represent the response to the manually delineated contours (“man”), by RUMC observers (“R”) and by UMCG observers (“G”), respectively. The last two bars represent the DLC results (in the same order).

**Supplementary figure 2:**
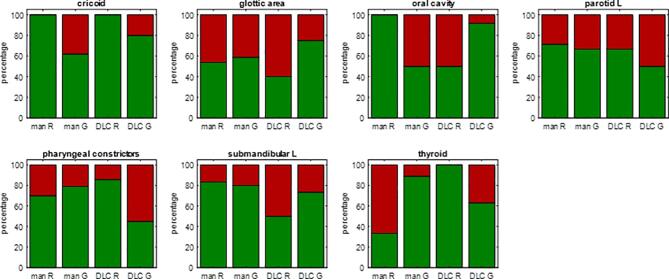


**Supplementary figure 3:**
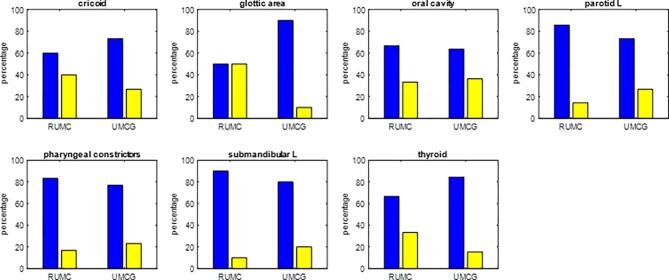


Considering the contour source, the RUMC observers correctly classified around 70% of all cases, for both manual and DLC contours (cf. Fig. 3A). The answers varied between OARS (e.g. cricoid mostly correct, glottic area more difficult to distinguish, cf. Supplementary Fig. S2). For the second question, the RUMC observers showed a large preference (80%) for manual contours when compared to DLC (cf. Fig. 3B). For individual OARs, this preference ranged from around 50% (glottic area) to 90% (left submandibular gland), cf. Supplementary Fig. S3. Finally, regarding the amount of editing, the RUMC observers accepted the manual contours in 79% of cases, while they approved of the DLC less often (53%, cf. Fig. 3C). Supplementary Fig. S4 shows large variations per OAR.

## 4 Discussion

In this study, an independent external validation of a DLC model was performed on a set of 58 HN cancer patients. DLC contours were evaluated by global and local geometric measures and a qualitative test. Reasonable and good correspondence was shown for the glandular OARs, mandible, brainstem and oral cavity, while the other aerodigestive tract OARs showed only moderate agreement. The largest local deviations were found caudally and/or cranially. The results demonstrate that the DLC model can provide a reasonable starting point for delineation when applied to an independent patient cohort.

Of course, the quantitative evaluation of the DLC on the external (RUMC) validation set should be considered in comparison to the results obtained with the original (UMCG) test set [13]. In that context, the glandular OARs (external DSC 0.78–0.83 vs. original 0.80–0.85) and the mandible in particular (DSC 0.90 vs. 0.95) showed comparable and good results. For the other OARs, especially those belonging to the aerodigestive tract, the scores for the external set were substantially lower (DSC 0.53–0.65) than those found originally (0.70–0.80). The oral cavity showed high DSC (0.85 vs. 0.91), but it should be noted that this is not a very sensitive measure for relatively large, round structures such as the oral cavity. The model itself hasn’t changed, therefore should be performing the same. Thus the only reasons for the lower quantitative scores as compared to Van Dijk et al. [13] can be a different input or a different reference.

The OARs that scored similar to the original test set in [13], also perform similar to other publications on this topic, such as the study by Liang et al. (mandible 0.91, parotids 0.85), who trained two CNNs on 186 nasopharynx patients [21]. The results are slightly better than those described by Ibragimov (parotid gland 0.78, submandibular gland 0.73), but those authors trained a CNN on fewer than 50 HN cases [22]. Like the model evaluated in this study, Van der Veen et al. also reported on a larger set of OARs [23]. They presented remarkably good results for the parotids and submandibular glands (DSC 0.91 to 0.97). However, these results might be overestimated because manual delineation was done with the automatic contours as a starting point. Their results on glottic and supraglottic areas and pharyngeal constrictor muscles were lower (and very comparable to the UMCG results).

The binned spatial evaluation proved very useful to identify the location of deviations, which were most often found in the caudal and/or cranial bins. This could be due to differences in input, e.g. partial volumes issues cause by CT slice thickness, as the average slice thickness used for the model training was smaller (2 mm) than the slice thickness of the external validation set (3 mm), while no resampling was done. This might have a large impact especially for structures like the glottic area, that occupy very few slices.

Apart from the input, another possible cause for the deviations between RUMC and UMCG results could lie in the reference; e.g. differences in manual delineation standards and interobserver variations between the two groups of observers. Although both centers used the same delineation guideline [14], there could be local variations in interpretation. To check for the latter, the qualitative evaluation using the Turing test done in [13] (i.e. using only the original UMCG data) was extended with RUMC observers.

Overall, the scores on source classification (around 70%) and preference for manual contours (almost 80%) were very similar between the two groups. There were some OAR-specific differences, with the RUMC observers choosing more often for the DLC than their UMCG colleagues for cricoid, thyroid and especially glottic area. The RUMC acceptance of the manual contours (delineated by UMCG observers) was overall even higher than the UMCG observers’ acceptance (79 vs. 68%). Admittedly the results of the qualitative evaluation are somewhat difficult to interpret, but there seemed to be no major disagreement on the delineation standards (although the slice-based qualitative evaluation did not allow assessment of the craniocaudal extent). RUMC DLC acceptance was a bit lower than showed by the UMCG observers (53 vs. 61%). This might be due to inexperience of the RUMC observers with DLC contours, making it more difficult to judge whether a deviation is clinically relevant.

The use of local (RUMC) clinical (uncurated) manual delineations as a reference is one of the limitations of this study. On the other hand, these contours are representative for clinical practice. Nevertheless, for further research, the contours should be checked and curated by a team of observers to ensure adherence to the guidelines. Prospectively acquiring multi-center data to an agreed imaging standard might also improve consistency.

In addition, the set of quantitative measures used could be improved and extended. It would be good to benchmark the measures, because while DSC is reasonably straightforward, HD results can be influenced substantially by the calculation algorithm. It would be good to include surface DSC [24] and/or Added Path Length [25], as these measures are a surrogate for the potential time saving of DLC in comparison to manual delineation. Valentini et al. [2] recommended to evaluate time saving, together with an assessment of dosimetric impact. Van Dijk et al. [13] already showed that a reduction in delineation time should be feasible and that dosimetric measures were not profoundly disturbed. Future work should also look to validate this externally.

Regarding the qualitative evaluation, the group of RUMC observers was small in comparison to the original group [13], and only the original UMCG test data were evaluated. In addition, the amount of observations per OAR was limited. Future work should comprise intergroup validation of manual delineation and the generation of a joint validation set, which could then be used for both quantitative and qualitative evaluations, to fully understand inter-institution variations. However, interobserver variability will remain a factor, as indicated by Van Dijk et al. [13], Van der Veen et al. [23], and Mattiucci et al. [26], who presented interobserver variabilities similar to the performance of the automatic delineation models for HN.

A generic and robust DLC model, trained to an agreed international standard, would be highly desirable. It is not practical and might not be desirable for each center to train their own model, because of the necessary expertise, time, and the required amount of high-quality curated manual delineations as training data. However, this will potentially require institutions to standardize not only contouring guidelines, but also image acquisition methods.

An alternative approach might be to re-train an existing model using a small training set of each center. Often, only a limited amount of data is available, insufficient to train a model from scratch. Transfer learning [9], [27], [28] is the method of using a pre-trained model to perform another task, after refinement with a small amount of new training data. Obviously, transfer learning for each center separately takes more time than using a generic model, but is faster than training a DLC model from scratch. In addition, only a small amount of data is necessary. However, each institution having a different model will reinforce inter-institution variations and guideline interpretation differences.

Whichever method is used, probably the goal should not be to obtain perfectly overlapping contours. This might also be nearly impossible in the HN region, involving a lot of small and complex structures. More important might be the evaluation of time saving/efficiency, and dosimetric effects [29]. With similar results on the parotid glands, oral cavity and mandible, both Van Dijk et al. [13] and Van der Veen et al. [23] described a more efficient delineation process using their automatic methods. Furthermore, Van Rooij et al. described that imperfect deep learning segmentation (also with similar DSC levels) does not necessarily result in inferior organ-at-risk dosimetry [30]. So even if the DLC results are not perfect, they can be used as input for manual editing and automated planning, provided they are carefully monitored.

This external validation demonstrated that the DLC model developed by Van Dijk et al. [13] can provide a reasonable starting point for delineation when applied to an independent patient cohort. Deviations found by the binned evaluation do not seem to be caused by local interpretations of delineation guidelines, but rather by interobserver variations and differences in image acquisition protocols. The use of a single model delineated to agreed international guidelines may help improve standardization between departments.

## Declaration of Competing Interest

The authors declare the following financial interests/personal relationships which may be considered as potential competing interests: University Medical Center Groningen has a research collaboration with Mirada Medical Ltd., Oxford, UK. Mirada Medical Ltd. has provided Radboud University Medical Center with the software for the external validation. Author MJG is an employee of Mirada Medical Ltd.

## Figures and Tables

**Supplementary figure 4:**